# Case Report: From multiple myeloma to plasmablastic lymphoma – a diagnostic dilemma in unraveling a rare transformation

**DOI:** 10.3389/fonc.2026.1778123

**Published:** 2026-03-10

**Authors:** Mohamad Shraim, Ahmad Salameh, Akram Karama, Mohaned Abu Lihya

**Affiliations:** 1 Faculty of Medicine, Al-Quds University, Abu Dis, Palestine; 2 Faculty of Applied Science, Palestine Ahliya University, Bethlehem, Palestine; 3 Department of Pathology, Beit Jala Governmental Hospital, Bethlehem, Palestine; 4 Department of Internal Medicine, Shamir Medical Center (Assaf Harofeh), Tzrifin, Israel; 5 Department of Hematology, Beit Jala Governmental Hospital, Bethlehem, Palestine; 6 Department of Pathology, Istishari Arab Hospital, Ramallah, Palestine

**Keywords:** case report, clonal evolution, DA-EPOCH chemotherapy, diagnostic challenge, EBV-negative PBL, extramedullary relapse, multiple myeloma transformation, plasmablastic lymphoma

## Abstract

**Introduction:**

Plasmablastic lymphoma (PBL) is a rare and aggressive subtype of diffuse large B-cell lymphoma. Its transformation from multiple myeloma (MM) is an exceptionally rare event, with only nine cases reported in the literature. Differentiating PBL from plasmablastic myeloma (PBM) is a significant diagnostic challenge due to overlapping morphological and immunophenotypic features, yet it is critical for determining the appropriate treatment regimen.

**Case presentation:**

We report the case of a woman in her 50s with a seven-year history of kappa-restricted MM who presented with a right leg mass. Biopsy confirmed a lambda-restricted plasmacytoma, indicating an extramedullary relapse. Two months after initiating therapy for myeloma relapse, she developed right inguinal lymphadenopathy. A lymph node biopsy revealed lambda-restricted plasmablasts positive for CD138, CD56, C-MYC, and Ki67 (100%), and negative for CD79a and EBER. In the absence of systemic MM-related end-organ damage and the presence of nodal disease, a diagnosis of PBL was favored over PBM.

**Conclusion:**

The patient was subsequently treated with DA-EPOCH chemotherapy (dose-adjusted etoposide, prednisone, vincristine, cyclophosphamide, doxorubicin), leading to significant regression of disease on interim PET scan. This case represents the 10th reported transformation of MM to PBL and highlights the diagnostic dilemma posed by these entities. It underscores the importance of clinical context, the potential for clonal evolution (evidenced by a light-chain switch), and the efficacy of lymphoma-specific chemotherapy in this setting.

## Highlights

- Distinguishing plasmablastic lymphoma (PBL) from plasmablastic myeloma (PBM) requires integration of immunohistochemistry (EBER, CD79a), clinical context (nodal vs. systemic involvement), and absence of myeloma-defining end-organ damage.
- DA-EPOCH chemotherapy may be preferable to CHOP for PBL arising from MM, given its efficacy in our case and PBL’s aggressive biology, though evidence remains limited.
- Autologous stem cell transplantation (HSCT) and radiotherapy warrant consideration in PBL, but their utility requires validation in larger studies.
- Clonal evolution (e.g., light-chain switch) may drive extramedullary relapse in MM, necessitating repeat biopsies to guide therapy.

## 1 Introduction

Multiple myeloma (MM) is an indolent plasma cell neoplasm, and patient outcomes have significantly improved due to novel agents and autologous stem cell transplantation. However, most patients relapse, with 30% of relapses occurring in extramedullary sites (1), some of which show plasmablastic features linked to plasmablastic myeloma (PBM). PBM cells are immature plasma cells with a round nucleus, nucleolus, and limited cytoplasm, expressing CD38 and CD138 with light chain restriction. PBM patients often show unfavorable features, including extensive bone marrow infiltration and high Ki67. In rare cases, multiple myeloma can transform into plasmablastic lymphoma (PBL), an aggressive lymphoma with plasmablastic features. Only nine cases of this transformation have been reported (2, 3) (**Table 1**).

**Table 1:** Reported cases of secondary plasmablastic tumors in patients with underlying plasma cell neoplasms (adapted from Colomo et al. (3)).

Case no./Source	Age/Sex	Site of PBL	Associated plasma cell disorder	EBV status	Key reported features
1 (Colomo et al. (3), Case 40)	54 M	Soft tissue, bone marrow	MM, s/p transplant	Negative	Listed as secondary plasmablastic tumor.
2 (Colomo et al. (3), Case 41)	59 M	Testicular	MM (previous)	Negative	Listed as secondary plasmablastic tumor.
3 (Colomo et al. (3), Case 43)	67 M	Lymph node	Vertebral plasmacytoma → MM (~4 years prior)	Positive	EBV+ nodal presentation.
4 (Colomo et al. (3), Case 44)	39 M	Soft tissues	Scapular plasmacytoma → MM (previous)	Negative	--
5 (Colomo et al. (3), Case 45)	55 F	Breast	MM (previous)	Negative	--
6 (Colomo et al. [., Case 46)	41 M	Skin, skull, bone marrow	MM (synchronous)	Negative	--
7 (Colomo et al. (3), Case 47)	77 M	Skin	MM (6 years prior)	Negative	--
8 (Colomo et al. (3), Case 48)	68 M	Bladder	MM (2 months prior)	Negative	--
9 (Colomo et al. (3), Case 42)	51 M	Lymph node	Nasal plasmacytoma (2 years prior)	Positive	EBV+ nodal presentation.
Present Case	F, 50s	Inguinal Lymph Node	Kappa-restricted MM (7 yrs prior)	Negative	First reported light-chain (κ→λ) switch; DA-EPOCH response.

PBL is a form of diffuse large B-cell lymphoma (DLBCL), first identified in the oral cavity of HIV-infected individuals, often with Epstein-Barr virus (EBV) co-infection. Its association with HIV and EBV is around 70% and 80%, respectively. The diagnosis of PBL has expanded to include HIV-negative, EBV-negative patients, often with immunosuppression. PBL originates from activated post-germinal center B-lymphocytes that develop a plasma cell-like phenotype, expressing markers like CD138, CD38, or MUM1 but lacking CD20 expression. Due to similar morphological and immunophenotypic features, distinguishing PBM from PBL can be difficult but is essential for appropriate treatment.

PBM is treated with anti-myeloma chemotherapy, including proteasome inhibitors, immunomodulatory agents, alkylating agents, CD38-targeting monoclonal antibodies, and autologous stem cell transplantation. In contrast, PBL is treated with combination chemotherapy and anti-lymphoma drugs (3–6).

## 2 Case presentation

A woman in her 50s with a seven-year history of kappa-restricted multiple myeloma (MM) presented with a two-month history of progressive swelling below her right knee. The patient had previously undergone autologous bone marrow transplantation and had shown a good response to treatment with Carfilzomib-Lenalidomide-Dexamethasone (KRD). She had also been maintained on lenalidomide and Zometa monthly for approximately 3 years prior to the development of the leg masses.

On physical examination, she had a soft, round mass slightly below her right knee. An initial extensive workup including complete blood count (CBC), serum calcium, creatinine, uric acid, lactate dehydrogenase (LDH), and serum and urine protein electrophoresis were all within normal limits. Serum free light chain analysis showed lambda and kappa light chain concentrations of 1.220 g/L and 1.788 g/L, respectively, with a kappa-lambda ratio of 1.465.

**Figure 1:**
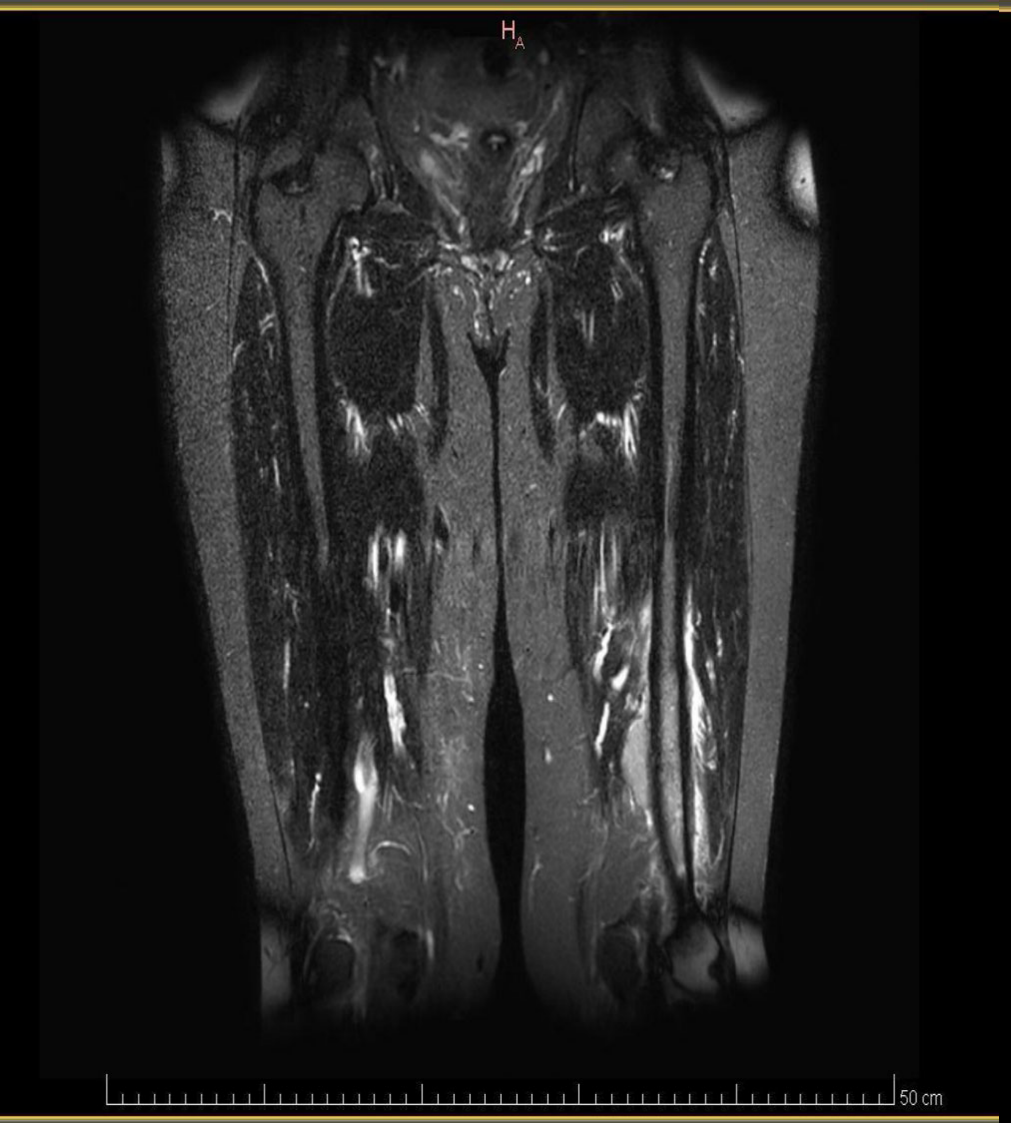
MRI of the legs showing a well-defined lobulated lesion on the left distal femur (arrow), suspicious for plasmacytoma.

**Figure 2:**
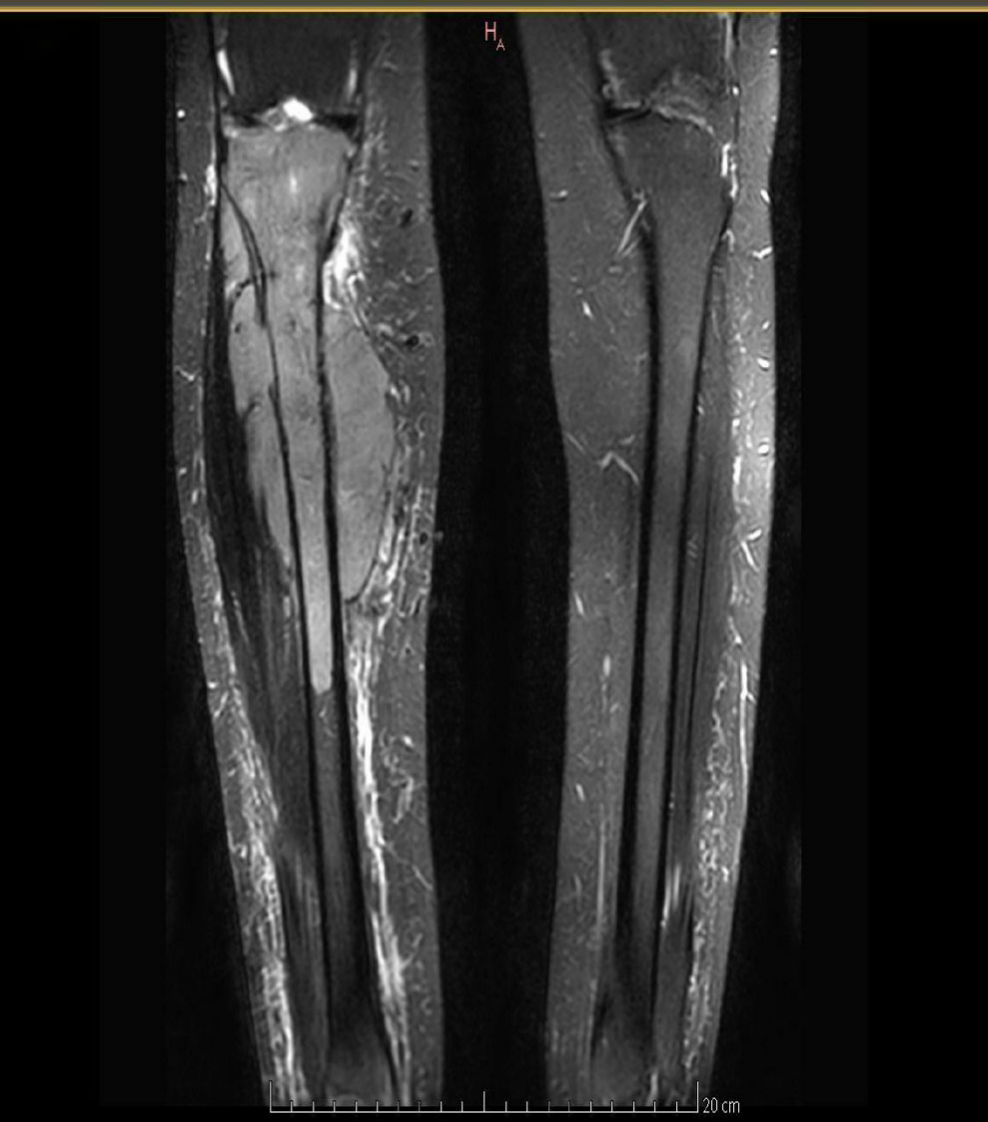
MRI of the legs showing a well-defined lobulated lesion on the right tibia (arrow), suspicious for plasmacytoma.

Magnetic resonance imaging (MRI) of both legs showed well-defined lobulated lesions on the left distal femur (**Figure 1**) and the right tibia (**Figure 2**), both of which were suspicious for plasmacytoma. MRI also revealed enlarged, pathological-appearing right inguinal lymph nodes that were deemed inaccessible for biopsy at that time. A biopsy was obtained from her right leg mass, which confirmed a lambda-restricted plasmacytoma, positive for CD138, CD56, and lambda immunostains.

Based on these findings, she was diagnosed with MM relapse in the form of a plasmacytoma. She was started on Pomalidomide-Cyclophosphamide-Dexamethasone (PCD), with a plan for radiotherapy.

**Figure 3:**
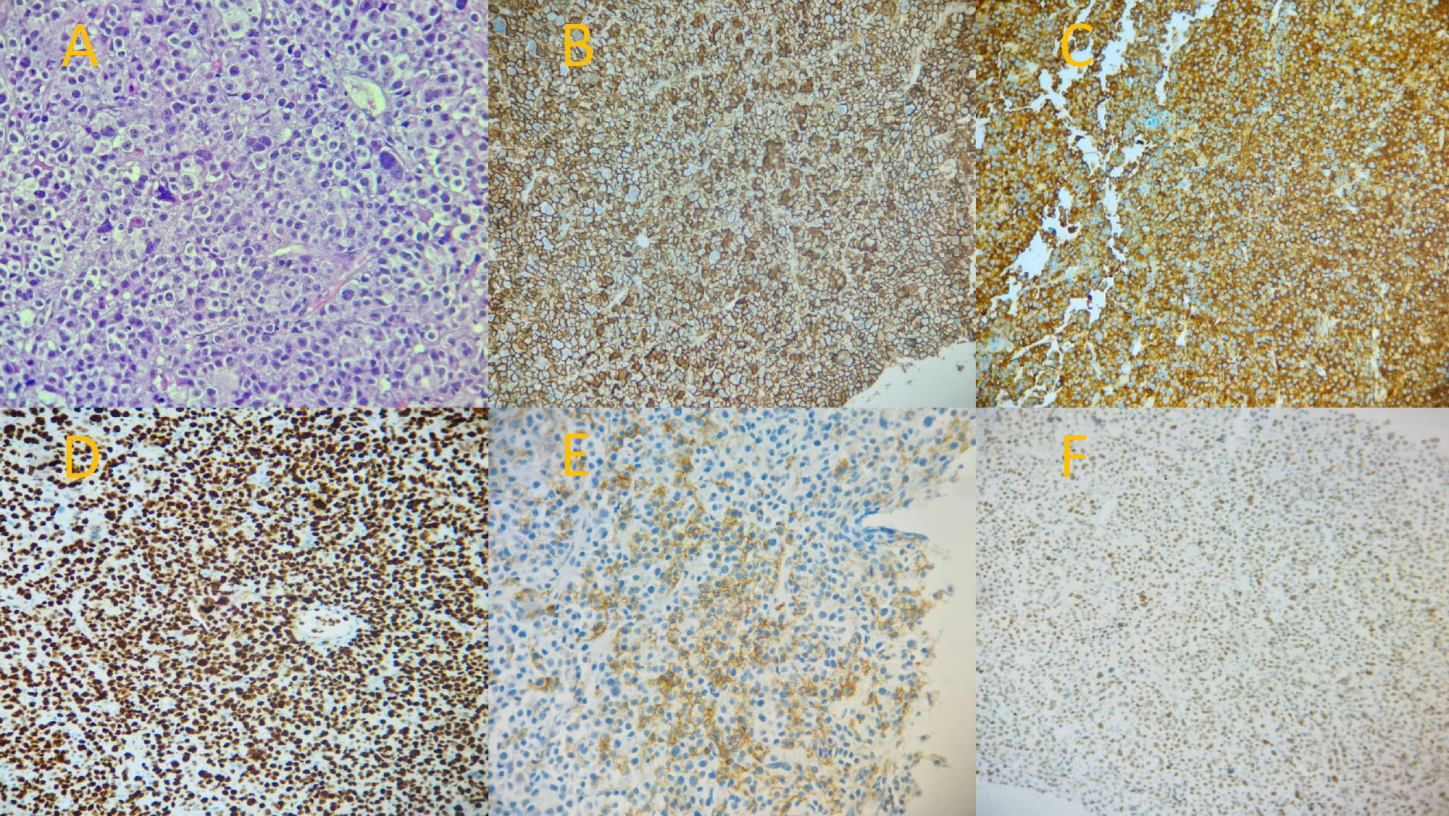
Histopathological and immunohistochemical examination of the right inguinal lymph node biopsy. **(A)** Hematoxylin and eosin (H&E) stain showing a diffuse infiltrate of plasmablasts with prominent nucleoli. **(B)** Positive CD138 immunohistochemical staining, confirming the plasma cell phenotype. **(C)** Diffusely positive lambda light chain immunohistochemical staining, demonstrating light chain restriction. **(D)** Ki67 immunohistochemical stain showing a proliferation index of nearly 100%, indicative of highly aggressive disease. **(E)** Positive CD56 immunohistochemical staining. **(F)** Positive C-MYC immunohistochemical staining.

Two months later, after one cycle of PCD, she complained of a new right groin swelling that progressively increased in size. Ultrasound revealed prominent right inguinal lymph nodes accessible for biopsy, and a core needle biopsy was performed. The results showed lambda-restricted plasmablasts, that were positive by immunohistochemistry for CD138, CD56, C-MYC, and Ki67 (100%), and negative for CD79a and Epstein-Barr virus-encoded RNA (EBER) (**Figure 3**). The prior laboratory workup was repeated with no significant changes. Additionally, bone marrow biopsy showed no morphological evidence of myeloma with 3% plasma cells.

The differential diagnosis for this patient was primarily plasmablastic lymphoma (PBL) versus plasmablastic myeloma (PBM). Based on the clinical features—particularly the presence of lymphadenopathy—and the absence of systemic MM-related end-organ damage (hypercalcemia, renal failure, anemia, or new lytic bone lesions), plasmablastic lymphoma was favored over plasmablastic myeloma.

The patient was started on DA-EPOCH therapy (dose-adjusted etoposide, prednisone, vincristine, cyclophosphamide, and doxorubicin). This led to dramatic improvements. PET scan results after 5 cycles showed resolution of previously seen hypermetabolic right pelvic and right inguinal lymph nodes. Additionally, there was significant regression of the right tibial lesion; however, a residual active process was still observed at the left femoral shaft. Further follow-up was recommended.

## 3 Discussion

PBL is subclassified as a diffuse large B cell lymphoma (DLBCL), despite its resemblance to plasma cell neoplasms; especially with its plasmablastic morphology, hence it expresses plasma cell differentiation markers (CD138, CD38, MUM1) and doesn’t express CD20. Based on a study on the genomic exploration of PBL, it was elucidated that its genomic alteration pattern appears to be more similar to DLBCL (AIDS- or non-AIDS-related) rather than PBM. Hence, at least at the genomic level, PBL is best classified as a subtype of DLBCL (7).

Our patient relapsed with a soft tissue lambda-restricted plasmacytoma and lambda-restricted plasmablastic lymphoma, involving the inguinal lymph nodes. Given the history of her kappa-restricted MM, this suggests a role of a subclone evolution in the mechanism of relapse. Based on a genomic study, new subclone emergence can be addressed to acquiring new lesions on the major clone or the survival of a subclone, that resisted treatment, even after the dominant clone has been eradicated (8).

The differential diagnosis was PBL vs PBM. Previous studies have showed an overlap between these two entities due to their similar morphologic and immunophenotypic features. Both share the expression of CD138, CD38, CD56, MUM1 and C-MYC; with EBER and CD79a supporting the diagnosis of PBL over PBM. The negativity of EBER and CD79a, however, does not rule out PBL nor confirm PBM, making the diagnosis more complicated. Likewise, our patient was positive for CD138, CD56, C-MYC and negative for CD79a and EBER. However, a systemic review of 76 cases showed that the association between PBL and EBV with negative-HIV was weaker (45%), in contrast to 75% coexistent rate in PBL with a positive HIV infection (6).

Given these findings, clinical and laboratory features were relied on to differentiate between PBL and PBM. In a previous systematic review of 16 patients, it was concluded that in the setting of negative EBER and CD79a, the presence of MM-caused end organ damage (hypercalcemia, renal failure, anemia and lytic bone lesions), paraproteinemia and bone marrow involvement support the diagnosis of PBM, while nodal involvement and the absence of the aforementioned systemic features favors the diagnosis of PBL (2). Despite our patient having several features overlapping between PBL and PBM, the absence of MM related end organ damage; coupled with the nodal involvement made PBL strongly supported.

To our knowledge, this case represents the 10th reported instance of multiple myeloma (MM) transforming into plasmablastic lymphoma (PBL) and the first to demonstrate: (1) a kappa-to-lambda light-chain switch during PBL transformation, and (2) EBV/HIV-negative PBL arising in an extramedullary site (inguinal lymph node) without systemic MM features. This case reinforces that clonal evolution can drive aggressive transformations in MM.

Given its rarity, there is no standard of care defining optimal therapeutic approach. The initial therapy has most often been cyclophosphamide, doxorubicin, vincristine, and prednisone (CHOP) or CHOP-like regimens. However, outcomes remain poor with a median overall survival (OS) of only about 14 months and a 5-year OS rate of 31% (9). Therefore, The National Comprehensive Cancer Network’s recommendations have supported the use of more intensive initial therapies, such as fractionated cyclophosphamide, vincristine, doxorubicin, and dexamethasone (hyper-CVAD) or dose-adjusted etoposide, prednisone, vincristine, and doxorubicin (DA-EPOCH). However, the evidence for a change in the outcomes has been conflicting (10).

The utility of other options is also controversial. Autologous hematopoietic stem cell transplantation (HSCT) was assessed in few case reports and was considered a feasible option in both consolidation of first line treatments and salvage therapy (11). While E. Tchernonog et al, reported in their cohort, that HSCT showed no significant benefit in OS and event free survival (EFS) (12). Radiotherapy is another option, which has been utilized in combination with chemotherapy for both consolidation and salvage. However, the contribution of radiotherapy to the outcomes couldn’t be determined, although studies suggested that it may have some positive results (6, 9, 13). Other options include, adding bortezomib to DA-EPOCH has shown efficacy in small case series (10). Rituximab also, has been added to chemotherapy, which has been showing good results, but the benefit remains uncertain (12).

The primary strength of this report lies in the detailed documentation of a rare transformation, notably the observed light-chain switch, which provides compelling clinical and immunophenotypic evidence for clonal evolution. The patient’s favorable initial response to a lymphoma-specific regimen (DA-EPOCH) offers valuable therapeutic insight for managing this diagnostic gray zone.

However, this study has several important limitations. As a single-case report, its conclusions regarding disease biology and optimal therapy cannot be generalized. The most significant limitation is the absence of molecular studies (e.g., *IGH* gene rearrangement or next-generation sequencing) to definitively prove clonal relatedness, as such testing is not available in our clinical setting. This reflects a common real-world diagnostic challenge and underscores the value of integrated clinicopathological correlation. Furthermore, while the protracted use of lenalidomide is a recognized risk factor for secondary primary malignancies—primarily solid tumors—an association with *de novo* plasmablastic lymphoma has not been established in the literature. Consequently, the hypothesis of clonal evolution remains the most plausible explanation for the transformation observed in this case.

## 4 Conclusion

This case highlights a rare and diagnostically challenging transformation of MM to PBL. It underscores the critical importance of integrating immunohistochemical results with clinical context to arrive at the correct diagnosis. The light-chain switch observed provides compelling evidence for clonal evolution as a mechanism of aggressive relapse. The successful initial response to DA-EPOCH in this case suggests that lymphoma-directed regimens are appropriate for PBL transformed from MM, though larger studies are needed to establish a standard of care.

## Data Availability

The raw data supporting the conclusions of this article will be made available by the authors, without undue reservation.
